# Lipoprotein(a) After Myocardial Infarction: Residual Risk, Measurement, and the Unresolved Therapeutic Question

**DOI:** 10.3390/biomedicines14102339

**Published:** 2026-10-14

**Authors:** Alexandra Gogan, Samuel Ardelean, Daniel Rus, Florina Rozina Căruntu, Bogdan-Flaviu Buz, Tudor Parvanescu, Minodora Andor

**Affiliations:** 1 Doctoral School of Medicine, “Victor Babeș” University of Medicine and Pharmacy of Timișoara, Eftimie Murgu Sq. No. 2, 300041 Timisoara, Romania; alexandra.gogan@umft.ro (A.G.); samuel.ardelean@umft.ro (S.A.); daniel.rus@umft.ro (D.R.); flaviu-bogdan.buz@umft.ro (B.-F.B.); 2 Department of Cardiology, Institute of Cardiovascular Diseases, Str. Gheorghe Adam No. 13A, 300310 Timisoara, Romania; 3 Department V—Internal Medicine 1, “Victor Babeș” University of Medicine and Pharmacy of Timișoara, Eftimie Murgu Square, No. 2, 300041 Timisoara, Romania; parvanescu.tudor@umft.ro (T.P.); andor.minodora@umft.ro (M.A.); 4 Multidisciplinary Heart Research Center, Faculty of Medicine, “Victor Babeș” University of Medicine and Pharmacy of Timișoara, Eftimie Murgu Square, No. 2, 300041 Timisoara, Romania

**Keywords:** Lipoprotein(a), myocardial infarction, coronary artery disease, residual cardiovascular risk, secondary prevention, lipid-lowering therapy, Lp(a)HORIZON, cardiovascular outcomes

## Abstract

Lipoprotein(a) [Lp(a)] is a highly heritable apoB-containing lipoprotein and a causal contributor to atherosclerotic cardiovascular disease that may contribute to residual cardiovascular risk after myocardial infarction (MI), while the clinical benefit of selective pharmacological Lp(a) lowering remains unresolved. This narrative review examines Lp(a) as a determinant of residual and recurrent cardiovascular risk after MI, integrating molecular biology, human genetics, coronary imaging, post-MI observational studies, measurement considerations, current management, and emerging therapies. Elevated Lp(a) has been associated with coronary plaque progression, high-risk plaque features, oxidized-phospholipid-related inflammatory pathways, recurrent ischemic events, and repeat revascularization, although prognostic associations vary across populations and endpoints. Because concentrations measured during acute MI may underestimate the stable Lp(a) phenotype in some patients, selective repeat measurement after clinical stabilization may be appropriate when classification could influence management or trial eligibility. Contemporary treatment remains centered on intensive control of LDL-C and other modifiable risk factors, while antisense oligonucleotides, small interfering RNAs, and oral particle-assembly inhibitors can produce profound Lp(a) reductions. According to sponsor-reported topline data released in September 2026, pelacarsen lowered Lp(a) but did not significantly reduce the prespecified primary cardiovascular endpoint in the overall Lp(a)HORIZON population; complete peer-reviewed results are not yet available. The sponsor-reported neutral HORIZON topline result raises unresolved questions regarding baseline Lp(a) and absolute reduction, timing of intervention and disease stage, and endpoint composition. Full peer-reviewed HORIZON data and ongoing cardiovascular outcome trials will be required to determine whether and under what conditions selective Lp(a) lowering translates into cardiovascular benefit.

## 1. Introduction—The Unresolved Residual Risk After Myocardial Infarction

### 1.1. Residual Cardiovascular Risk Despite Contemporary Secondary Prevention

Major advances in reperfusion, antithrombotic therapy, lipid lowering, and comprehensive secondary prevention have substantially improved outcomes after myocardial infarction (MI). Nevertheless, survivors remain at considerable risk of recurrent cardiovascular events despite guideline-directed therapy and intensive low-density lipoprotein cholesterol (LDL-C) lowering. This persistent burden has shifted attention toward determinants of residual cardiovascular risk that are not fully captured by conventional lipid measurements. Recent participant-level evidence further supports that Lp(a)-associated cardiovascular risk is not simply a reflection of LDL-C-mediated risk [1].

The 2019 ESC/EAS dyslipidaemia guideline established intensive LDL-C lowering as a central component of secondary prevention and recognized Lp(a) measurement as a means of identifying individuals with substantial inherited lipid-related risk [2]. The 2023 ESC guideline for acute coronary syndromes subsequently reinforced early initiation and intensification of evidence-based secondary-prevention therapy following MI [3]. These recommendations provide the clinical framework within which Lp(a) should be interpreted: as an additional risk determinant rather than a substitute for established post-MI treatment.

Lipoprotein(a) [Lp(a)] is an LDL-like apoB-100-containing particle covalently linked to apolipoprotein(a) [apo(a)]. Its concentration is predominantly genetically determined and is relatively stable under clinically stable conditions. Contemporary European and North American guidance therefore supports Lp(a) measurement at least once during adulthood, with concentrations around or above 50 mg/dL generally considered clinically relevant, although molar thresholds differ between guideline frameworks [4,5,6,7]. Cardiovascular risk increases continuously across the Lp(a) distribution rather than beginning at a single biological threshold.

Earlier European and national consensus documents helped establish this continuous-risk concept and emphasized that very high Lp(a) concentrations may confer a lifetime cardiovascular burden comparable with that of other major inherited lipid disorders [8,9]. The more recent recommendations used throughout this review therefore represent an evolution of a longstanding evidence base rather than a newly recognized association.

The relevance of elevated Lp(a) may be particularly important in secondary prevention, where absolute cardiovascular risk is already high. In 1131 patients with acute coronary syndrome undergoing emergency percutaneous coronary intervention and treated with statins at discharge, Takahashi et al. found that higher Lp(a) was associated with a greater risk of major adverse cardiac events after adjustment for conventional cardiovascular risk factors [10], suggesting a contribution to residual risk despite contemporary treatment.

### 1.2. Why Lp(a) Belongs in the Post-MI Residual-Risk Discussion

Post-MI studies support Lp(a) as a residual-risk marker, although not uniformly. Cao et al. found an independent association with subsequent cardiovascular events in 3864 patients with previous MI [11], and Dai et al. reported a similar association after acute MI [12]. In contrast, Park et al. found that an unadjusted association was attenuated after multivariable adjustment [13].

A 2025 meta-analysis of 23 cohorts comprising 30,027 patients confirmed a modest but significant association between higher Lp(a) and post-MI major adverse cardiovascular events, with heterogeneity according to follow-up, clinical phenotype, and endpoint definition [14].

These prognostic data should be interpreted at the level of individual outcome components. In several post-MI cohorts, the association with composite MACE was driven largely by repeat or unplanned revascularization, whereas associations with recurrent MI and cardiovascular or all-cause mortality were less consistent [14].

### 1.3. The Unresolved Therapeutic Question

Lp(a)HORIZON was designed as the first large randomized cardiovascular outcome trial directly testing selective Lp(a) lowering. The phase III study enrolled 8323 patients with established cardiovascular disease and Lp(a) ≥ 70 mg/dL and compared pelacarsen 80 mg administered subcutaneously every four weeks with placebo on top of guideline-directed therapy [15].

On 4 September 2026, sponsor-reported topline results indicated that pelacarsen lowered Lp(a) but did not meet the prespecified primary cardiovascular endpoint in the overall trial population [16]. At the time of writing, complete peer-reviewed results—including detailed treatment-effect estimates, absolute event rates, achieved Lp(a) reductions, individual endpoint components, and comprehensive subgroup analyses—have not yet been reported.

The HORIZON announcement sharpens the central question of this review. Because the available information is limited to sponsor-reported topline data, it does not identify the reasons for the neutral primary endpoint or determine whether treatment effects differed across prespecified subgroups or individual endpoint components. Any explanation must therefore remain hypothesis-generating until the complete randomized dataset is available.

In light of this, this review integrates molecular biology, human genetics, cardiovascular imaging, post-MI observational evidence, measurement studies, current management, and forthcoming pharmaceutical trials to explore Lp(a) following myocardial infarction. The timing and interpretation of Lp(a) measurement following acute MI, recurrent cardiovascular risk, and the implications of HORIZON for present and upcoming Lp(a)-targeted outcome trials are all given special attention.

Accordingly, the purpose of this review is not to provide another comprehensive overview of Lp(a), but to address questions that are specific to the post-MI setting. These include the interpretation and timing of Lp(a) measurement after acute infarction, the phenotype of recurrent cardiovascular risk associated with elevated Lp(a), the implications of Lp(a) for contemporary post-MI management, the extent to which randomized post-ACS evidence informs Lp(a)-related risk reduction, and how the design of ongoing outcome trials should be interpreted from a secondary-prevention perspective.

### 1.4. Literature Search Strategy

This narrative review was developed using a structured literature search of PubMed/MEDLINE, Scopus, Web of Science, ClinicalTrials.gov, and major professional-society guideline and consensus documents. Searches covered database inception through 10 September 2026. Search terms were used alone and in combination and included “lipoprotein(a)”, “Lp(a)”, “apolipoprotein(a)”, “myocardial infarction”, “acute coronary syndrome”, “secondary prevention”, “recurrent cardiovascular events”, “MACE”, “PCI”, “plaque progression”, “vulnerable plaque”, “oxidized phospholipids”, “inflammation”, “thrombosis”, “fibrinolysis”, “measurement”, “assay”, “pelacarsen”, “olpasiran”, “lepodisiran”, “zerlasiran”, “muvalaplin”, “Lp(a)HORIZON”, “OCEAN(a)”, “ACCLAIM-Lp(a)”, and “MOVE-Lp(a)”. Priority was given to peer-reviewed human studies, randomized trials, meta-analyses, large prospective cohorts, contemporary imaging studies, and recent guidelines or consensus statements, particularly publications from 2021–2026. Older landmark epidemiological, genetic, and mechanistic studies were retained when they established concepts essential to interpretation of the contemporary evidence. Studies were selected for direct relevance to Lp(a) biology, causality, post-MI prognosis, measurement, current management, or targeted therapeutic development. Duplicate reports, isolated case reports, and publications without direct relevance to the scope of the review were not prioritized. Because the objective was a narrative synthesis rather than an exhaustive systematic review or meta-analysis, no protocol was prospectively registered, and no PRISMA flow diagram was used. Evidence was synthesized thematically, with particular emphasis on distinguishing causal association, prognostic information, pharmacological target engagement, and randomized cardiovascular outcome validation.

This narrative review has several limitations. Since it was a narrative synthesis, neither a formal risk-of-bias assessment nor a prospectively registered systematic-review process served as the basis for research selection. Despite the use of a controlled literature search, narrative-review bias may have affected the evidence’s selection and interpretation. Furthermore, the therapeutic landscape is changing quickly, especially in light of Lp(a)HORIZON’s September 2026 topline announcement, for which full peer-reviewed findings were not yet available at the time of writing. As comprehensive HORIZON data and results from current cardiovascular outcome trials become available, conclusions about treatment efficacy, subgroup effects, and the clinical implications of targeted Lp(a) lowering should be reviewed.

## 2. Lipoprotein(a): Biology and Genetic Basis Relevant to Cardiovascular Disease

Lipoprotein(a) [Lp(a)] is an apoB-containing lipoprotein composed of an LDL-like particle covalently linked to apolipoprotein(a) [apo(a)]. Apo(a) contains a variable number of kringle IV type 2 repeats, which contributes to marked interindividual differences in apo(a) isoform size and circulating Lp(a) concentration [6,17,18]. Lp(a) concentrations are determined predominantly by hepatic production and genetic variation within the *LPA* locus and remain relatively stable across the life course [6,19,20].

Lp(a) combines the atherogenic properties of an apoB-containing particle with additional biological effects related to apo(a) and oxidized phospholipids (OxPL). OxPL associated with Lp(a) may promote inflammatory signaling, endothelial activation, and vascular-cell responses, although the relative contributions of cholesterol deposition, apo(a), OxPL, and other particle-associated mechanisms remain incompletely separated in humans [21,22].

The strong genetic determination of Lp(a), including associations with KIV-2 copy number and *LPA* variants such as rs10455872 and rs3798220, provides the biological basis for lifelong exposure to elevated Lp(a) and underpins subsequent causal analyses [23,24]. These features are particularly relevant after myocardial infarction because the same inherited exposure persists despite treatment of the culprit coronary lesion.

## 3. Why Could Lp(a) Matter Particularly After Myocardial Infarction?

After myocardial infarction, residual cardiovascular risk arises from both the treated culprit lesion and persistent disease throughout the coronary circulation. Elevated Lp(a) may be relevant through several pathways, but the strength of evidence is not uniform. Evidence is most consistent for atherogenicity, coronary plaque progression, and adverse plaque characteristics [25,26,27,28,29,30,31]. Evidence for clinically important thrombotic or antifibrinolytic effects [32,33,34,35,36] and for a direct role in post-MI myocardial inflammation [37] remains less definitive and is derived mainly from mechanistic, translational, or associative studies.

### 3.1. Coronary Plaque Progression

Serial coronary imaging has strengthened the association between Lp(a) and progression of atherosclerotic plaque. Kaiser et al. showed that higher Lp(a) was associated with accelerated progression of coronary atheroma despite contemporary preventive therapy [25]. More recently, Nurmohamed et al. linked elevated Lp(a) with long-term plaque progression, low-density plaque burden, and pericoronary inflammatory changes on coronary computed tomography angiography [26].

A 2026 systematic review and meta-analysis including 16 imaging studies and 19,822 participants further supported associations between higher Lp(a) and greater coronary plaque burden and high-risk plaque characteristics [27]. Separate meta-analytic evidence has also evaluated the relationship between Lp(a) and coronary artery calcification, extending Lp(a) imaging phenotyping beyond noncalcified and high-risk plaque [28]. Contemporary cohort data have also evaluated the combined relationship of Lp(a), coronary artery calcium, and ASCVD [29]. Although study populations, imaging modalities, and definitions varied, the overall evidence suggests that Lp(a) may contribute to atherosclerotic progression beyond risk captured by LDL-C alone.

In the post-MI patient, this is clinically relevant because untreated nonculprit plaques remain exposed to the same lifelong Lp(a)-related biology after successful treatment of the culprit lesion. Continued plaque progression may provide one substrate for recurrent coronary events.

### 3.2. Lp(a) and the Vulnerable Plaque Phenotype

The PROSPECT II substudy provides particularly relevant evidence after MI. Three-vessel coronary imaging was performed in 865 patients with recent MI after treatment of flow-limiting lesions [30]. Whereas LDL-C and related lipid measures were associated with overall pancoronary plaque burden, Lp(a) was associated more specifically with focal untreated vulnerable plaques characterized by high plaque burden and high lipid content.

The pattern raises the possibility that conventional LDL-related pathways predominantly influence the global extent of atherosclerosis, whereas Lp(a) may contribute disproportionately to selected high-risk lesions. More recent IVUS data have similarly associated higher Lp(a) with plaque vulnerability and compensatory vascular enlargement [31], consistent with the broader imaging meta-analysis [27]. Whether Lp(a) lowering can reverse these plaque characteristics remains unknown. Despite this post-MI imaging rationale, the major selective Lp(a)-lowering outcome trials have not tested treatment initiation during the index ACS hospitalization or the immediate post-infarction period. In Lp(a)HORIZON, for example, qualifying myocardial infarction had to have occurred at least 3 months before screening and randomization. Whether selective Lp(a) lowering initiated earlier after ACS could influence vulnerable nonculprit plaque biology or recurrent ischemic events therefore remains untested [38].

### 3.3. Oxidized Phospholipids and Vascular Inflammation

Lp(a) is a major carrier of oxidized phospholipids, which can be present within the particle and covalently associated with apo(a) [21,22]. This provides a plausible mechanism through which Lp(a) may influence inflammatory activity in addition to its role as an apoB-containing lipoprotein.

Van der Valk et al. showed that individuals with elevated Lp(a) had increased arterial wall inflammation and an activated monocyte phenotype; experimental attenuation of OxPL-related effects reduced several proinflammatory responses [39]. The study provides one mechanistic link between Lp(a)-associated OxPL and vascular inflammation.

Systemic inflammatory biomarkers tell a less straightforward story. In OCEAN(a)-DOSE, profound Lp(a) lowering with olpasiran reduced OxPL associated with apoB-containing particles without parallel reductions in high-sensitivity C-reactive protein or interleukin-6 [40]. Recent human studies have further examined the interaction between elevated Lp(a), inflammatory state, and coronary disease risk [41]. Meta-analytic evidence also indicates that the association between elevated Lp(a) and cardiovascular events persists across different hs-CRP strata [42]. Circulating Lp(a), OxPL, local vascular inflammation, and systemic inflammatory markers may therefore capture partly distinct biological processes.

### 3.4. Thrombosis, Fibrinolysis, and Platelet Biology

The structural homology between apo(a) and plasminogen has long raised the possibility of antifibrinolytic effects. Interactions with plasminogen binding, fibrin clot dissolution, platelets, and coagulation pathways have been proposed [32,33]. In post-MI patients with highly elevated Lp(a), *LPA* haplotypes have also been associated with parameters of coagulation, fibrinolysis, and inflammation [34]. Evidence for these effects is less definitive than the evidence linking Lp(a) with atherosclerotic coronary disease.

Recent mechanistic work by Clark et al. showed that Lp(a) can prolong ex vivo clot-lysis time through effects related to clot formation and fibrin architecture [35]. The study also emphasized that effects observed with isolated apo(a) cannot automatically be extrapolated to the intact circulating Lp(a) particle.

Clinical observations are directionally supportive but remain associative. In younger patients with STEMI, elevated Lp(a) has been associated with greater culprit-artery thrombus burden [36], while experimental and translational data suggest interactions between Lp(a) and platelet function [32]. At present, Lp(a) is best described as having potential prothrombotic and antifibrinolytic properties rather than an established clinical hypercoagulable mechanism. Importantly, these mechanistic observations do not currently justify modification of post-MI antithrombotic therapy solely on the basis of an elevated Lp(a) concentration. The choice and duration of antiplatelet or anticoagulant therapy should continue to follow established indications and individualized ischemic and bleeding-risk assessment rather than Lp(a) level [3].

### 3.5. Lp(a), OxPL, and Myocardial Inflammation After Acute MI

An emerging question is whether Lp(a)-related biology after MI extends beyond the coronary plaque to the infarcted myocardium. Atallah et al. measured Lp(a), OxPL-apoB, and OxPL-apo(a) during hospitalization for acute MI and at 30 days, together with serial 18F-fluorodeoxyglucose positron-emission tomography assessment of myocardial inflammatory activity [37]. Higher Lp(a), OxPL-apoB, and OxPL-apo(a) at 30 days were associated with greater myocardial inflammatory activity, and changes in Lp(a) and OxPL-apoB correlated with changes in myocardial 18F-FDG uptake.

The study links Lp(a)-related pathways with persistent tissue-level inflammation during the early post-MI period, but the association cannot establish that Lp(a) directly impairs myocardial healing or that lowering Lp(a) improves post-infarction remodeling.

### 3.6. Integrating the Post-MI Mechanisms

Taken together, the mechanistic evidence is strongest for continued atherogenic lipid deposition, coronary plaque progression, and an association with high-risk plaque phenotypes [25,26,27,28,29,30,31]. Oxidized-phospholipid-related vascular inflammation provides additional biological plausibility [39,40,41,42]. By comparison, the clinical relevance of proposed thrombotic and antifibrinolytic effects [32,33,34,35,36] and the association with post-MI myocardial inflammation [37] remain less firmly established.

These pathways should not be viewed as mutually exclusive. The clinical phenotype associated with elevated Lp(a) may reflect the cumulative interaction of atherosclerotic progression, inflammatory signaling, plaque vulnerability, and potentially arterial thrombosis over decades of genetically determined exposure.

Most mechanistic and imaging evidence remains observational or translational. Associations with adverse plaque or inflammatory phenotypes do not show that pharmacological lowering reverses these processes or prevents events. The subsequent sections therefore turn from biological plausibility to causal evidence and direct therapeutic testing.

The principal mechanistic pathways linking elevated Lp(a) to residual cardiovascular risk after myocardial infarction are summarized in Figure 1.

## 4. From Epidemiological Association to Causality

The evidence linking elevated lipoprotein(a) [Lp(a)] to atherosclerotic cardiovascular disease has progressed from epidemiological association to a strong body of human genetic evidence. Human genetics has been particularly informative because elevated Lp(a) is not simply a biomarker accompanying conventional cardiovascular risk factors: lifelong exposure to higher concentrations appears to contribute causally to coronary disease and myocardial infarction.

### 4.1. Epidemiological and Genetic Evidence

Large prospective cohorts have demonstrated a continuous association between higher Lp(a) concentrations and coronary cardiovascular risk, including myocardial infarction [43,44,45,46,47]. However, the strongest evidence supporting causality comes from human genetics. *LPA* variants associated with higher Lp(a), including rs10455872 and rs3798220, are also associated with greater coronary disease risk, whereas genetic variants associated with lower Lp(a) concentrations are associated with lower risk [23,48,49,50].

Mendelian-randomization analyses further support a causal relationship between lifelong Lp(a) exposure and coronary disease. Burgess et al. demonstrated an approximately linear association between genetically predicted differences in Lp(a) and coronary risk and estimated that relatively large absolute differences in lifelong Lp(a) exposure may be required to produce cardiovascular effects comparable with LDL-C lowering [51]. More recent genetic analyses have reinforced that Lp(a)-related coronary risk is at least partly distinct from LDL-C-mediated risk [52,53].

Together, epidemiological and genetic evidence strongly supports elevated Lp(a) as a causal contributor to coronary atherosclerotic disease and myocardial infarction. However, causal inference from lifelong genetic exposure does not establish that pharmacological lowering initiated later in life will produce an equivalent reduction in cardiovascular events [51].

### 4.2. Lifetime Exposure Versus Pharmacological Lowering

The distinction between lifelong exposure and later pharmacological intervention is central to interpreting Lp(a) as a therapeutic target. Individuals genetically predisposed to lower Lp(a) experience reduced exposure over decades, whereas participants in cardiovascular outcome trials generally begin treatment only after clinically manifest atherosclerotic disease has developed. Baseline and on-treatment Lp(a) remain associated with cardiovascular events in statin-treated populations, but these observational associations cannot define the magnitude or duration of pharmacological lowering required to reproduce the protection associated with lifelong genetically lower exposure [54].

Population-based analyses have estimated that substantial absolute Lp(a) reductions may be required to achieve clinically meaningful reductions in recurrent cardiovascular events [55]. Such estimates are not therapeutic thresholds, but they emphasize why baseline concentration, absolute reduction, and duration of treatment are important when interpreting Lp(a)-lowering outcome trials.

## 5. Lipoprotein(a) and Recurrent Cardiovascular Risk After Myocardial Infarction

After myocardial infarction, the clinical relevance of lipoprotein(a) [Lp(a)] shifts from prediction of a first atherosclerotic event toward its potential contribution to residual and recurrent cardiovascular risk. This question is particularly relevant because post-MI patients already receive intensive secondary prevention. Within this setting, elevated Lp(a) may identify a subgroup in whom substantial risk persists despite treatment of conventional risk factors.

### 5.1. Recurrent Major Adverse Cardiovascular Events

Several observational studies have associated elevated Lp(a) with recurrent events after previous or recent MI. In a prospective cohort of 3864 patients with previous MI, Cao et al. demonstrated that higher Lp(a) was independently associated with subsequent cardiovascular events during a median follow-up of 4.1 years [11]. The composite included recurrent MI, ischemic stroke, and cardiac mortality, supporting the persistence of Lp(a)-related risk after coronary disease has become clinically manifest.

The overall evidence was subsequently synthesized by Liu et al. in a 2025 meta-analysis of 23 cohorts comprising 30,027 patients after acute MI. Higher Lp(a) was associated with increased risk of major adverse cardiovascular events both when analyzed categorically and continuously [14]. The pooled association was modest overall and became more pronounced during follow-up exceeding one year. However, MACE definitions varied among studies and frequently included coronary revascularization; the pooled result should therefore not be interpreted as equally consistent evidence for recurrent MI or mortality.

This temporal difference may be relevant because early post-MI outcomes are strongly influenced by infarct severity, ventricular dysfunction, acute procedural complications, and early treatment, whereas later events may increasingly reflect progression of systemic atherosclerotic disease and persistent exposure to residual risk factors.

Data from Nishiyama et al. provide additional support [56]. Among 868 patients with STEMI, elevated Lp(a), defined using a study-derived threshold of 47.26 mg/dL, independently predicted a composite of cardiovascular death, nonfatal MI, nonfatal stroke, or any revascularization. However, the excess risk was driven predominantly by subsequent revascularization, illustrating why the composition of MACE endpoints must be considered when interpreting post-MI Lp(a) studies.

Elevated Lp(a) appears to mark recurrent cardiovascular risk after MI, but the composition of that risk is important. In several cohorts, the excess composite-event rate was driven predominantly by repeat or unplanned coronary revascularization, while associations with recurrent MI, cardiovascular death, and all-cause mortality were less consistent [56,57,58,59,60,61,62].

### 5.2. Reinfarction and Cardiovascular Mortality

Recurrent MI provides a particularly relevant endpoint because it more directly reflects recurrent ischemic coronary disease than broader composite outcomes.

Miñana et al. evaluated patients after acute MI over a median follow-up of approximately 9.9 years [57]. Higher Lp(a) was positively and nonlinearly associated with the total burden of recurrent infarction, whereas no significant association was observed with all-cause mortality. The long duration of follow-up is particularly informative because it supports a potential relationship between persistent Lp(a) exposure and recurrent coronary events over many years.

A similar distinction emerged in routine clinical data reported by Šuran et al. [58]. Among 2248 patients hospitalized with AMI, Lp(a) > 90 mg/dL was independently associated with recurrent MI, while no significant association with cardiovascular or all-cause mortality was demonstrated in the overall cohort. A stronger association with reinfarction was observed among older women, although this subgroup finding should be considered hypothesis-generating.

The meta-analysis by Liu et al. further demonstrated that relationships with individual outcome components were less uniform than the association with composite MACE [14].

Across these studies, the prognostic signal appears more closely tied to recurrent atherosclerotic and ischemic events than to mortality, which is influenced by a wider range of cardiac and noncardiac determinants.

### 5.3. Post-PCI Events and Repeat Revascularization

Because contemporary MI management frequently involves percutaneous coronary intervention (PCI), the association between Lp(a) and long-term outcomes after coronary intervention is highly relevant.

Xu et al. evaluated 4469 patients with previous cardiovascular events undergoing PCI and followed them for approximately five years [59]. Lp(a) ≥ 30 mg/dL independently predicted major adverse cardiac and cerebrovascular events, with associations also reported for all-cause and cardiac mortality.

In an ACS cohort of 765 patients, Yang et al. similarly found elevated Lp(a) to be associated with recurrent cardiovascular events [60]. However, among the individual components, the clearest relationship was with unplanned revascularization rather than recurrent MI, stroke, or mortality.

Nishiyama et al. also reported higher rates of subsequent revascularization among STEMI patients with elevated Lp(a), involving both de novo and restenotic lesions [56]. More recent PCI data have also linked elevated Lp(a) with subsequent coronary revascularization [61]. These observations are compatible with imaging evidence linking Lp(a) with coronary plaque progression and high-risk plaque phenotypes.

The broader evidence was summarized in a 2025 systematic review and meta-analysis of 14 studies involving 40,241 patients after PCI [62]. Elevated Lp(a) was associated with higher rates of MACE, myocardial infarction, cardiovascular death, all-cause death, and stroke during a mean follow-up of approximately 4.9 years.

Elevated Lp(a) also appears to mark residual risk after PCI [63]. Revascularization, however, is not equivalent to spontaneous MI or cardiovascular death: it is influenced by symptom burden, surveillance, restenosis, clinician decision-making, and progression of previously untreated lesions. This matters when revascularization contributes substantially to a composite endpoint.

### 5.4. Persistent and Cumulative Lp(a) Exposure

Most prognostic studies rely on a single Lp(a) measurement, but emerging longitudinal evidence has examined whether persistent or cumulative exposure carries additional information.

Wang et al. studied 1131 patients with AMI in whom Lp(a) was measured at admission and again one year later [64]. Compared with patients whose Lp(a) remained low, persistent elevation was independently associated with a higher risk of MACCE, cardiovascular death, nonfatal stroke, unplanned revascularization, and all-cause death during approximately 50 months of follow-up.

A subsequent study involving 2634 patients with AMI evaluated cumulative Lp(a) burden over time [65]. During a median follow-up of 55.2 months, both higher Lp(a) and greater cumulative burden were associated with MACCE, cardiovascular death, nonfatal MI, and nonfatal stroke. The association was particularly pronounced for recurrent nonfatal MI.

The longitudinal studies raise a biologically plausible possibility: cardiovascular risk may reflect not only whether Lp(a) crosses a threshold, but also the magnitude and duration of exposure.

Longitudinal changes still require cautious interpretation. Although Lp(a) is predominantly genetically determined, measured concentrations can vary with acute illness, analytical variability, renal or inflammatory conditions, and other clinical factors. An observational measure of “cumulative Lp(a)” is therefore not identical to lifelong exposure estimated from human genetics.

This longitudinal perspective also makes the timing of post-MI Lp(a) measurement clinically relevant, a question addressed in the next section.

### 5.5. Heterogeneity and Potential Effect Modifiers

A consistent feature of the post-MI literature is heterogeneity in the strength of the association between Lp(a) and recurrent events.

Diabetes illustrates this particularly well. Li et al. studied 1543 patients with STEMI undergoing emergency PCI [66]. Among patients with diabetes, Lp(a) ≥ 50 mg/dL was associated with increased MACE risk compared with Lp(a) <10 mg/dL, whereas a similar association was not demonstrated among patients without diabetes.

Silverio et al. reported the opposite interaction [67]. Among 1018 patients after MI, elevated Lp(a) predicted the composite of recurrent MI and all-cause death in patients without diabetes, whereas no significant association was observed in those with diabetes.

These discordant findings should be interpreted cautiously because the two studies differed in population, Lp(a) categorization, and endpoint definition. Taken together, they do not support a consistent diabetes-specific modification of Lp(a)-related recurrent cardiovascular risk.

Other potential modifiers have also been reported. The Liu meta-analysis suggested differences according to sex, diabetes, hypertension, MI phenotype, and duration of follow-up [14], while Šuran et al. observed a particularly strong recurrent-MI signal among women older than 65 years [58]. Such findings require external confirmation before being used for clinical risk stratification or treatment selection.

More broadly, heterogeneity across post-MI studies may arise from differences in Lp(a) assays and units, concentration thresholds, timing of measurement, ancestry, extent of coronary disease, renal function and diabetes, background lipid-lowering treatment, duration of follow-up, and endpoint composition.

Ancestry- and sex-related differences deserve particular attention when post-MI cohorts are compared. Lp(a) distributions vary markedly among ancestral groups, and a fixed absolute threshold may identify different proportions of each population [46,47,68]. Evidence in women also suggests that the association may depend on the concentration range and the accompanying lipid profile [69]. Sex-specific analyses from ODYSSEY OUTCOMES have further examined the interplay between sex, Lp(a), and cardiovascular outcomes after acute coronary syndrome [70]. Future post-MI studies should therefore report sex-stratified results, ancestry, assay characteristics, and both absolute Lp(a) values and prespecified concentration categories.

The post-MI literature supports elevated Lp(a) as a marker of residual cardiovascular risk, with the most reproducible signal involving progression of coronary disease and repeat revascularization. Associations with recurrent MI and mortality have been reported, but they are less uniform across cohorts and meta-analyses. The strength of association varies by population, endpoint definition, and follow-up, so a single Lp(a) value should not be treated as a deterministic predictor.

Timing also affects interpretation. Concentrations obtained during acute myocardial infarction may not always represent the patient’s stable Lp(a) phenotype, which motivates the measurement considerations discussed next. Representative studies evaluating the association between Lp(a) and recurrent cardiovascular outcomes after myocardial infarction or coronary intervention are summarized in Table 1.

## 6. When Should Lipoprotein(a) Be Measured After Acute Myocardial Infarction?

Because lipoprotein(a) [Lp(a)] concentrations are predominantly genetically determined, a reliable measurement obtained under stable clinical conditions generally does not require routine repetition. Acute myocardial infarction (AMI) may justify selective reassessment because concentrations obtained during the acute event can underestimate those observed after clinical stabilization. This distinction supports selective repeat measurement in clinically relevant situations, not routine serial testing after every infarction.

### 6.1. Lp(a) During Acute Myocardial Infarction Versus the Stable Phase

In clinically stable populations, Lp(a) demonstrates high long-term reproducibility. In the UK Biobank, Trinder et al. evaluated 16,017 individuals with repeated measurements separated by a median of 4.42 years and found a strong correlation between baseline and follow-up concentrations (Spearman rho = 0.96) [71]. These data support the concept that a single reliable measurement is generally sufficient under stable conditions.

The acute MI setting may differ. Ziogos et al. evaluated 108 patients with STEMI or NSTEMI in whom Lp(a) was measured within 24 h of admission and again six months later [72]. Median Lp(a) increased from 53.5 nmol/L during hospitalization to 58.0 nmol/L at follow-up, with substantial interindividual variability.

A similar pattern was observed in the STEMI-Lipids Study [73]. Among 86 patients with STEMI, median Lp(a) increased from 7.9 mg/dL at presentation to 8.4 mg/dL on day 1, 9.3 mg/dL on day 2, and 11.2 mg/dL at 3–6 months. Short-term changes in Lp(a) have also been observed during the first days after acute coronary syndrome, suggesting that the acute-phase trajectory itself may contain prognostic information [74].

More recently, Tamehri Zadeh et al. specifically examined temporal changes in Lp(a) after AMI [75]. In 173 patients with AMI and Lp(a) ≥ 75 nmol/L, median Lp(a) increased from approximately 214 nmol/L during the acute event to 249 nmol/L after stabilization, corresponding to a median increase of 16.5%. Importantly, the stable measurement showed a stronger relationship with the underlying *LPA* genetic risk score than the acute value.

Measurements obtained during AMI may therefore underestimate the patient’s stable Lp(a) phenotype. The practical implication is selective repeat measurement after clinical stabilization when an acute-phase result could alter risk classification, cascade screening, clinical-trial eligibility, or future treatment decisions; it is not routine retesting for every post-MI patient.

### 6.2. Should Lp(a) Be Remeasured After Myocardial Infarction?

Repeat testing is unlikely to add substantial information when a reliable Lp(a) measurement has already been obtained under stable clinical conditions. Likewise, a clearly elevated value measured during AMI is unlikely to lose its qualitative significance after recovery.

The potential value of repeat testing is greatest when the initial concentration is close to a clinically relevant threshold. Harb et al. evaluated 609 individuals with repeated measurements and found that 38.1% experienced an absolute change of at least 10 mg/dL [76]. Among participants whose initial Lp(a) was 30–50 mg/dL, approximately 53% were reclassified into another category after repeat testing.

Similar findings were reported by Awad et al. in 11,669 individuals [77]. Patients with clearly low or high initial values usually remained within the same category, whereas approximately half of those initially in the intermediate range changed category. Variability has also been observed among placebo-treated participants in OCEAN(a)-DOSE [78]. A 2026 study further documented measurable intraindividual variability in serial Lp(a) concentrations [79].

Selective repeat measurement is most useful when the acute-phase result could change clinical classification or management. Reassessment after stabilization may be particularly relevant when:

• Lp(a) was measured for the first time during the acute infarction;
• The initial value is close to a clinically relevant threshold;
• The result appears discordant with the patient’s clinical or family phenotype;
• Classification may influence cascade screening;
• a specific concentration threshold may determine eligibility for a clinical trial or future Lp(a)-directed therapy.

No universally validated interval for repeat measurement has been established. Post-MI studies have generally reassessed Lp(a) after approximately 3–6 months [72,73,75], making post-stabilization retesting reasonable when the result could materially alter interpretation or management rather than as a routine fixed-time procedure.

### 6.3. Measurement Units and Assay Standardization

Interpretation of Lp(a) is complicated by the marked structural heterogeneity of apo(a). Variable KIV-2 copy number produces apo(a) isoforms of different molecular sizes, which can introduce bias into immunoassays whose antibody response depends on isoform size.

Contemporary recommendations therefore favor assays that minimize sensitivity to apo(a) isoform size and are traceable to internationally recognized reference materials [5,6,80]. Recent interlaboratory comparisons nevertheless demonstrate that clinically relevant differences between contemporary Lp(a) assays persist [81].

Foundational analytical studies demonstrated substantial method-dependent variation when assays with different sensitivities to apo(a) size were compared [82]. Introduction of the WHO/IFCC reference reagent SRM 2B improved calibration and comparability, but standardization does not eliminate all isoform-related bias or make mass and molar units interchangeable [82]. Reports should therefore identify the assay, unit, calibration approach, and, when available, evidence of isoform insensitivity [83].

Lp(a) may be expressed as mass concentration, typically in mg/dL, or as molar particle concentration, typically in nmol/L. These measurements are not directly interchangeable because the molecular mass of Lp(a) varies according to apo(a) isoform size. Consequently, a universal conversion factor between mg/dL and nmol/L should not be applied to individual patients [6,80,84]. Recent analyses from the Lp(a)HORIZON program have further demonstrated the limitations of assuming direct concordance between mass- and molar-based Lp(a) measurements [85].

This limitation is also important when comparing observational studies and clinical trials, which frequently use different assays, units, and eligibility thresholds. Apparently similar cutoffs expressed in mg/dL and nmol/L should therefore not be assumed to represent exact biological equivalents.

A further interpretive issue is that conventionally measured LDL-C includes cholesterol carried within Lp(a). Analyses using estimated Lp(a)-cholesterol-corrected LDL-C show that correction can alter apparent LDL-C levels and treatment-related comparisons, particularly at high Lp(a) concentrations [86]. Because the cholesterol content of individual Lp(a) particles is variable, however, fixed correction formulas remain approximations and should not be used to infer an exact patient-specific LDL-C value.

### 6.4. Clinically Relevant Thresholds

Cardiovascular risk associated with Lp(a) increases continuously across the concentration distribution rather than beginning abruptly at a single threshold.

The 2025 ESC/EAS focused update identifies Lp(a) > 50 mg/dL and >105 nmol/L within its respective reporting framework as clinically relevant cardiovascular risk modifiers [4]. These values should not be interpreted as mathematically interchangeable.

The 2024 National Lipid Association update proposes a practical classification [80]:

• <30 mg/dL or <75 nmol/L: lower risk;
• 30–50 mg/dL or 75–125 nmol/L: intermediate range;
• ≥50 mg/dL or ≥125 nmol/L: high risk.

The 2026 ACC/AHA guideline similarly considers ≥50 mg/dL or ≥125 nmol/L elevated [5].
**Context/Source****Threshold**ESC/EAS 2025>50 mg/dL; >105 nmol/LNLA 2024<30 mg/dL/<75 nmol/L low; 30–50/75–125 intermediate; ≥50/≥125 highACC/AHA 2026≥50 mg/dL or ≥125 nmol/LLp(a)HORIZON≥70 mg/dL; prespecified ≥90 mg/dL subgroupOCEAN(a)-Outcomes≥200 nmol/LACCLAIM-Lp(a)≥175 nmol/LThresholds are presented in the units used by the respective guideline or trial and should not be interpreted using a fixed conversion factor between mg/dL and nmol/L.

The different molar values paired with the commonly used 50 mg/dL threshold reflect distinct guideline frameworks, not exact mathematical conversions. This is another reason to avoid fixed conversion between mass and molar units.

After MI, an acute-phase value just below a clinical threshold deserves particular caution because Lp(a) may rise after stabilization. The threshold itself should also remain a risk-classification aid rather than a rigid biological boundary, since cardiovascular risk is continuous.

### 6.5. A Practical Post-MI Measurement Framework

Accordingly, acute myocardial infarction should not be interpreted as an indication for routine serial Lp(a) testing in all patients. A reliable measurement obtained under clinically stable conditions generally does not require repetition. However, when Lp(a) is first measured during the acute infarction and the result is borderline or close to a clinically relevant threshold, selective reassessment after clinical stabilization is reasonable if it could influence risk classification, family screening, clinical-trial eligibility, or future management.

## 7. Current Management of the Post-MI Patient with Elevated Lipoprotein(a)

The identification of elevated lipoprotein(a) [Lp(a)] after MI raises an immediate therapeutic question: what can currently be done to reduce the associated residual cardiovascular risk? Although elevated Lp(a) is strongly linked to ASCVD, no selective Lp(a)-lowering pharmacological strategy has yet demonstrated definitive cardiovascular outcome benefit. Current management therefore focuses on intensive reduction in modifiable cardiovascular risk, particularly aggressive lowering of low-density lipoprotein cholesterol (LDL-C), while recognizing Lp(a) as a largely inherited residual-risk factor.

### 7.1. Intensive LDL-C Lowering

Elevated Lp(a) adds to the rationale for intensive LDL-C lowering after MI. Because conventional outcome-proven therapy does not substantially lower Lp(a), reducing other modifiable apoB-containing lipoproteins becomes especially important [4,5,6,80].

The 2025 ESC/EAS focused update emphasizes intensive management of cardiovascular risk factors in individuals with elevated Lp(a), while the 2026 ACC/AHA dyslipidemia guideline similarly recommends early and optimal control of modifiable risk in patients with Lp(a) ≥ 50 mg/dL or ≥125 nmol/L [4,5].

For patients with established ASCVD at very high risk, including many post-MI patients, intensive statin therapy remains foundational, with ezetimibe and/or a PCSK9 monoclonal antibody added when necessary to achieve recommended lipid targets [5].

In practice, an elevated Lp(a) concentration is an additional reason to minimize exposure to modifiable atherogenic lipoproteins; it is not an argument against further LDL-C lowering.

### 7.2. Statins and Ezetimibe

Statins remain the cornerstone of lipid-lowering therapy after MI irrespective of Lp(a) concentration. Their cardiovascular benefit in secondary prevention is firmly established, even though they do not meaningfully lower Lp(a).

The effect of statins on Lp(a) has been debated. Earlier analyses suggested a modest increase in Lp(a) in some individuals receiving statins [87], whereas a more recent meta-analysis of randomized lipid-lowering trials found no significant overall effect [88]. Differences between studies may reflect drug type, baseline Lp(a), assay methodology, and study population.

Regardless of these differences, the clinical implication is clear: a possible modest increase in Lp(a) is not a reason to avoid, reduce, or discontinue statin therapy. Contemporary recommendations continue to support intensive statin treatment in high-risk patients with elevated Lp(a) [4,5].

Ezetimibe similarly has little or no clinically meaningful effect on Lp(a) [88]. Its role in this population is therefore not direct Lp(a) lowering, but additional reduction in LDL-C and overall apoB-containing lipoprotein burden.

### 7.3. PCSK9 Inhibition and Residual Lp(a)-Related Risk

PCSK9 monoclonal antibodies occupy a distinctive position because they combine profound LDL-C lowering with moderate reductions in circulating Lp(a). Across randomized trials, evolocumab and alirocumab reduce Lp(a) by approximately 20–30% on average [88,89,90].

In FOURIER, Lp(a) was measured in 25,096 patients with established ASCVD [89]. Higher baseline concentrations predicted coronary events independently of LDL-C, and evolocumab reduced Lp(a) by a median of 26.9%. Patients with higher baseline Lp(a) appeared to derive greater absolute coronary benefit from evolocumab.

The FOURIER analysis cannot isolate Lp(a) lowering as the cause of benefit because evolocumab simultaneously produced substantial LDL-C reduction.

Similar observations emerged from ODYSSEY OUTCOMES, which is particularly relevant because the trial enrolled patients after recent acute coronary syndrome. In a post hoc analysis, higher baseline Lp(a) predicted a greater burden of subsequent cardiovascular events, while reductions in both Lp(a) and corrected LDL-C during alirocumab treatment were independently associated with fewer total cardiovascular events [91].

Schwartz et al. further showed that among patients whose LDL-C was already relatively well controlled, the clinical benefit of alirocumab appeared more evident when residual Lp(a) remained elevated [92].

More recent post hoc analyses from ODYSSEY OUTCOMES suggest that baseline Lp(a) may also influence the timing of cardiovascular benefit with alirocumab after recent ACS. In patients with baseline Lp(a) ≥ 30 mg/dL or ≥50 mg/dL, the treatment hazard ratio and 95% confidence interval for MACE remained below 1 from approximately 1.31 and 1.25 years after randomization, respectively [93]. These findings are clinically relevant to the post-ACS setting, but they should not be interpreted as proof that Lp(a) lowering itself mediated the benefit, because alirocumab simultaneously produced substantial LDL-C reduction.

The PCSK9 data are consistent with Lp(a) contributing to residual lipid-related risk and make these agents attractive in high-risk patients with elevated Lp(a). Their proven clinical role, however, rests on LDL-C lowering and cardiovascular outcome benefit, not on validation as Lp(a)-targeted therapy.

### 7.4. Comprehensive Residual-Risk Modification

Because Lp(a) is largely genetically determined, lifestyle measures do not substantially lower it. Its presence still strengthens the case for comprehensive secondary prevention, including control of lipids, blood pressure, diabetes, smoking, body weight, physical activity, and adherence to evidence-based therapy [5,6,80].

Beyond conventional lipid targets, readily available lipid-derived indices may capture complementary cardiometabolic risk in other high-risk cardiovascular populations; for example, an elevated triglyceride-to-HDL cholesterol ratio has been associated with increased ischemic stroke risk in patients with paroxysmal atrial fibrillation [94].

The importance of treatment adherence is also illustrated by clinical reports describing the development of coronary disease after discontinuation of preventive cardiovascular therapy, although such observations should be regarded as illustrative rather than causal evidence [95].

### 7.5. Family Screening

Because Lp(a) is highly heritable, markedly elevated values after MI should prompt consideration of screening in first-degree relatives, particularly after premature MI or when disease severity appears disproportionate to conventional risk factors [80].

### 7.6. Lipoprotein Apheresis

Lipoprotein apheresis can substantially reduce circulating Lp(a) and remains available in selected patients with progressive cardiovascular disease according to jurisdiction-specific criteria [80,96]. Observational studies and registries have reported lower cardiovascular event rates after initiation of apheresis [97,98,99,100], but these before-and-after data are susceptible to confounding and do not provide randomized evidence that Lp(a) lowering itself improves cardiovascular outcomes. Its invasive nature, resource requirements, and limited availability restrict its role to highly selected patients.

### 7.7. The Current Therapeutic Gap

Current management of elevated Lp(a) after MI remains indirect.

Statins and ezetimibe substantially reduce LDL-C but do not meaningfully lower Lp(a). PCSK9 monoclonal antibodies provide proven cardiovascular benefit and moderate Lp(a) reduction, but existing analyses cannot determine how much of that benefit is attributable specifically to Lp(a).

For now, management centers on minimizing modifiable cardiovascular risk while recognizing elevated Lp(a) as an important source of residual risk. The limitations of conventional therapy also explain the development of antisense oligonucleotides, small interfering RNAs, and oral agents that target the Lp(a) pathway more directly [101].

## 8. Targeted Lipoprotein(a)-Lowering Therapies

The development of therapies capable of selectively and profoundly lowering lipoprotein(a) [Lp(a)] has transformed the therapeutic landscape. Unlike conventional lipid-lowering agents, emerging compounds directly target apolipoprotein(a) synthesis or formation of the mature Lp(a) particle. These strategies include antisense oligonucleotides, small interfering RNAs (siRNAs), and oral small-molecule inhibitors.

Phase 2 trials have demonstrated Lp(a) reductions ranging from approximately 70% to more than 90%, establishing that Lp(a) is pharmacologically modifiable. However, the neutral topline result of Lp(a)HORIZON emphasizes that biomarker reduction and cardiovascular outcome benefit are distinct endpoints. Emerging therapies should therefore be compared not only by percentage Lp(a) reduction, but also by mechanism, absolute reduction, durability, dosing, target population, and ultimately cardiovascular outcomes.

Several pharmacological strategies now achieve substantial reductions in circulating Lp(a). Pelacarsen, a hepatocyte-directed antisense oligonucleotide, produced dose-dependent reductions of approximately 35–80% in phase 2 testing [102]. Small interfering RNA approaches have generally produced deeper and more sustained suppression: olpasiran reduced Lp(a) by approximately 70–100% in OCEAN(a)-DOSE [103], lepodisiran achieved placebo-adjusted time-averaged reductions of up to approximately 94% in ALPACA [104], and zerlasiran produced reductions of approximately 81–86% through 36 weeks [105].

Muvalaplin represents a mechanistically distinct oral strategy that inhibits formation of the intact Lp(a) particle; in the KRAKEN trial, placebo-adjusted reductions reached approximately 86% at the highest tested dose [106]. These studies demonstrate that Lp(a) is pharmacologically modifiable through antisense therapy, RNA interference, and inhibition of particle assembly.

However, biomarker reduction and cardiovascular outcome benefit are distinct endpoints. The sponsor-reported neutral topline result of Lp(a)HORIZON reinforces the need to interpret emerging agents according to baseline Lp(a), absolute and sustained reduction, treatment timing, patient population, and ultimately randomized cardiovascular outcomes [15,16]. The principal pharmacological and outcome-trial characteristics of the major agents are summarized in Table 2.

## 9. Lp(a)HORIZON: The First Major Test of the Therapeutic Lp(a) Hypothesis

### 9.1. Why Lp(a)HORIZON Was Needed

By the beginning of the current decade, epidemiological and human genetic evidence strongly supported elevated lipoprotein(a) [Lp(a)] as a causal contributor to coronary disease and myocardial infarction [51]. What remained unanswered was a therapeutic question: can selective Lp(a) lowering initiated after cardiovascular disease has been established reduce subsequent events?

Pelacarsen made this question experimentally testable: phase 2 data showed Lp(a) reductions of up to approximately 80% [102], while genetic and population analyses suggested that large absolute reductions might be required for clinically meaningful benefit [51,55].

Lp(a)HORIZON was designed to answer that question in a randomized cardiovascular outcome trial.

### 9.2. Study Design and Population

Lp(a)HORIZON (NCT04023552) was a phase III, randomized, double-blind, placebo-controlled cardiovascular outcomes trial enrolling 8323 patients with established cardiovascular disease and Lp(a) ≥ 70 mg/dL [15,38].

Eligible participants had previous myocardial infarction, ischemic stroke, or symptomatic peripheral artery disease. They were randomized to pelacarsen 80 mg administered subcutaneously every four weeks or placebo in addition to contemporary standard-of-care cardiovascular therapy [15].

Importantly, HORIZON was not an exclusively post-MI trial, although myocardial infarction was the predominant qualifying event, accounting for 6786 of 8323 participants (81.5%). Ischemic stroke and symptomatic peripheral artery disease were also qualifying conditions. A prespecified or exploratory analysis restricted to participants with prior MI would therefore be highly relevant to the post-MI setting; however, such a subgroup analysis should be interpreted together with formal interaction testing and cannot supersede the neutral primary result in the overall randomized population.

The prespecified primary endpoint was a four-component MACE consisting of cardiovascular death, nonfatal myocardial infarction, nonfatal stroke, or urgent coronary revascularization requiring hospitalization. The primary analysis included both the overall population with Lp(a) ≥ 70 mg/dL and a prespecified subgroup with Lp(a) ≥ 90 mg/dL [15]. The event-driven trial was planned to continue until 993 adjudicated primary events had accrued.

### 9.3. The September 2026 Sponsor-Reported Topline Result

On 4 September 2026, Novartis announced sponsor-reported topline results indicating that Lp(a)HORIZON did not meet its primary cardiovascular endpoint [16]. Pelacarsen lowered circulating Lp(a), but treatment did not demonstrate a statistically significant reduction in the prespecified primary composite endpoint compared with placebo in the overall trial population.

Lp(a)HORIZON is the first completed phase III randomized trial designed to test whether selective pharmacological lowering of Lp(a) can reduce cardiovascular events in patients with established cardiovascular disease. Interpretation is necessarily constrained by the information currently available. The complete results have not yet appeared in a peer-reviewed manuscript, and detailed hazard ratios and confidence intervals, absolute event rates, achieved Lp(a) concentrations, absolute and percentage reductions, individual endpoint components, adherence data, and comprehensive subgroup analyses have not yet been reported. Until these data are available, conclusions should remain limited to the sponsor-reported failure of the primary endpoint in the overall population.

The prespecified higher-Lp(a) subgroup is especially relevant because higher baseline concentrations permit larger absolute reductions for a given percentage effect. Threshold-dependent conclusions should wait for the full subgroup data and formal interaction analyses.

### 9.4. What HORIZON Does—And Does Not—Establish

According to the sponsor-reported topline announcement, pelacarsen did not significantly reduce the prespecified primary cardiovascular endpoint in the overall HORIZON population [16]. The announcement does not yet permit detailed conclusions regarding effect magnitude, individual endpoint components, or prespecified subgroups.

The result also cannot yet be generalized to every Lp(a)-lowering strategy. Ongoing programs differ in mechanism, depth and durability of reduction, baseline Lp(a) thresholds, populations, and endpoint definitions.

Several of these issues had already been recognized before HORIZON was completed. Malick et al. highlighted baseline Lp(a), population selection, expected treatment effect, follow-up duration, and endpoint composition as important considerations in the design of Lp(a)-lowering cardiovascular outcome trials [110].

HORIZON is best viewed as the first major randomized test of selective Lp(a) lowering in patients with established cardiovascular disease. The sponsor reported a neutral primary endpoint, but full peer-reviewed data are required before the result can be interpreted beyond that high-level conclusion.

The announcement therefore raises three principal questions regarding interpretation and future trial design: baseline Lp(a) and the magnitude of absolute reduction, timing of intervention and disease stage, and endpoint composition. These questions are discussed in Section 10.

## 10. Questions Raised by the Lp(a)HORIZON Topline Result

The sponsor-reported neutral topline result of Lp(a)HORIZON raises important questions regarding the translation of Lp(a) lowering into cardiovascular benefit [16]. However, because the complete peer-reviewed results are not yet available, the factors underlying the neutral primary endpoint remain unknown. The following considerations should therefore be viewed as questions relevant to interpretation and future trial design rather than established explanations for the trial result.

### 10.1. Baseline Lp(a) and the Magnitude of Absolute Reduction

The magnitude of Lp(a) lowering may need to be considered in absolute as well as relative terms. Genetic and population analyses have suggested that relatively large absolute reductions in Lp(a) may be required to translate into clinically meaningful cardiovascular benefit [51,55]. Thus, the same percentage reduction may represent substantially different absolute changes depending on the baseline concentration.

This makes baseline Lp(a) particularly relevant when interpreting outcome trials. HORIZON enrolled patients with Lp(a) ≥ 70 mg/dL and included a prespecified analysis among participants with Lp(a) ≥ 90 mg/dL [15]. Data from the higher-Lp(a) subgroup, together with achieved concentrations and absolute reductions, will therefore be important when the complete trial results become available.

Indirect evidence from PCSK9 inhibitor trials also suggests that higher baseline Lp(a) identifies greater residual cardiovascular risk [89,91,92]. However, because these agents substantially lower LDL-C in addition to Lp(a), these analyses cannot establish a treatment threshold for selective Lp(a) lowering.

### 10.2. Timing of Intervention and Disease Stage

A second question concerns the timing of intervention. Mendelian randomization reflects differences in Lp(a) exposure acting over decades, whereas pharmacological treatment in HORIZON was initiated after established cardiovascular disease had already developed [15,51]. These two forms of exposure modification are therefore not directly equivalent.

Patients with previous myocardial infarction, ischemic stroke, or peripheral artery disease may already have accumulated substantial atherosclerotic burden before selective Lp(a) lowering is initiated [15]. Observational studies after acute myocardial infarction have also associated persistent and cumulative Lp(a) exposure with subsequent cardiovascular events [64,65].

Whether earlier initiation or longer treatment duration would produce a different cardiovascular effect remains unknown. The available topline HORIZON information does not allow treatment timing or disease stage to be identified as an explanation for the neutral primary endpoint.

### 10.3. Endpoint Composition and Trial Design

Endpoint composition is another important consideration in Lp(a)-lowering outcome trials. HORIZON used a four-component composite of cardiovascular death, nonfatal myocardial infarction, nonfatal stroke, and urgent coronary revascularization requiring hospitalization [15]. These outcomes are clinically important but may not have identical relationships with Lp(a)-mediated risk.

Endpoint selection had already been identified as an important issue in the design of Lp(a)-lowering cardiovascular outcome trials [110]. Post-MI observational studies have shown relatively consistent associations of elevated Lp(a) with recurrent ischemic events and revascularization, whereas associations with mortality have been less uniform [11,14,56,57,58,59,60,61,62].

The complete HORIZON report should therefore be examined at the level of both the primary composite and its individual components. Nevertheless, exploratory differences among individual components cannot override a neutral primary endpoint [16].

At present, none of these considerations can explain the neutral topline result. Full peer-reviewed HORIZON data are required to determine whether baseline Lp(a), achieved absolute reduction, treatment timing, or endpoint composition materially influenced the observed outcome [16]. These issues should therefore be regarded as questions for interpretation and future trial design rather than explanations for the trial result.

The distinction between lifelong causal exposure and late pharmacological intervention, together with the unresolved questions raised by HORIZON, is summarized in Figure 2.

## 11. Beyond HORIZON: The Next Tests of the Lp(a) Hypothesis

The neutral topline result of Lp(a)HORIZON makes the remaining cardiovascular outcome trials more informative, not less. Their designs differ in therapeutic mechanism, magnitude and durability of Lp(a) reduction, baseline threshold, disease stage, and endpoint composition.

The ongoing cardiovascular outcome trials provide complementary tests of the therapeutic Lp(a) hypothesis because they differ in mechanism, baseline Lp(a) threshold, disease stage, and endpoint composition. OCEAN(a)-Outcomes (NCT05581303) enrolled 7297 participants with established coronary ASCVD and Lp(a) ≥ 200 nmol/L and evaluates olpasiran using a coronary-focused primary endpoint of coronary heart disease death, myocardial infarction, or urgent coronary revascularization [107]. Compared with HORIZON, it therefore tests a different therapeutic platform, a higher entry Lp(a) threshold, and a more coronary-specific outcome structure.

ACCLAIM-Lp(a) (NCT06292013) is evaluating lepodisiran in approximately 17,300 participants with Lp(a) ≥ 175 nmol/L, including both established ASCVD and selected high-risk individuals without a previous major cardiovascular event [108]. MOVE-Lp(a) (NCT07157774) similarly includes both secondary- and high-risk primary-prevention populations and tests the oral particle-assembly inhibitor muvalaplin in approximately 10,450 participants with Lp(a) ≥ 175 nmol/L [109].

OCEAN(a)-PreEvent (NCT07136012) extends the olpasiran program to approximately 11,000 participants without a previous major atherothrombotic event and with Lp(a) ≥ 200 nmol/L [107]. This trial addresses an earlier stage of disease than HORIZON or OCEAN(a)-Outcomes and may therefore help clarify whether intervention before a first major event differs from treatment initiated after established ASCVD.

Taken together, these trials will test whether the clinical effect of Lp(a) lowering depends on therapeutic mechanism, baseline concentration, absolute and sustained reduction, disease stage, or endpoint selection. Until their results are available, phase 2 studies establish pharmacological target engagement but not cardiovascular outcome efficacy [102,103,104,105,106]. The principal design differences among the major cardiovascular outcome trials are summarized in Table 3.

The combined trial program should clarify which features, if any, determine clinical benefit: mechanism, depth and duration of lowering, absolute reduction, baseline concentration, stage of disease, or endpoint selection.

Until those results are available, phase 2 studies establish that Lp(a) can be lowered pharmacologically; they do not establish that doing so prevents cardiovascular events. The field has therefore moved from the question of feasibility to the harder questions of timing, patient selection, and clinical efficacy.

## 12. Clinical Perspective: What Should the Cardiologist Do Now?

The expanding evidence surrounding lipoprotein(a) [Lp(a)] creates a practical challenge. Elevated Lp(a) identifies increased atherosclerotic cardiovascular risk, but selective pharmacological lowering has not yet shown definitive cardiovascular outcome benefit. Following the sponsor-reported neutral HORIZON topline result, clinical practice has to separate information that is actionable now from interventions that remain investigational [16].

In the post-MI patient, Lp(a) currently functions primarily as an inherited residual-risk marker that can influence intensity of prevention, family screening, interpretation of recurrent risk, and eligibility for emerging therapeutic trials.

### 12.1. Who Should Have Lp(a) Measured After Myocardial Infarction?

Contemporary European and North American recommendations support Lp(a) measurement at least once during adulthood [4,5,6]. Accordingly, a patient presenting with MI should have Lp(a) measured if a reliable previous result is not already available [111]. Despite these recommendations, implementation in routine post-ACS care remains limited. In a 2026 real-world analysis of 231,996 patients hospitalized with acute coronary syndrome and treated with PCI who had no previous documented Lp(a) measurement, only 2.5% underwent Lp(a) testing within the following 12 months, highlighting a substantial gap between guideline recommendations and clinical practice [112]. The Brussels International Declaration has further emphasized broader implementation of Lp(a) testing and management in routine clinical practice [113].

In secondary prevention, the purpose differs from primary risk prediction. A patient who has experienced MI already has established ASCVD; Lp(a) measurement therefore identifies an additional inherited component of residual risk rather than determining whether cardiovascular risk is present.

Measurement is particularly informative in premature MI, recurrent coronary events despite appropriate treatment, a strong family history of premature ASCVD, or unexpectedly extensive coronary disease [114]. These features increase the value of testing but should not be prerequisites for it.

Lp(a) ≥ 50 mg/dL is widely considered elevated, although molar thresholds differ between guideline frameworks. ESC/EAS uses approximately >105 nmol/L, whereas ACC/AHA and NLA use ≥125 nmol/L [4,5,80]. These values should be interpreted as clinical risk thresholds rather than exact mathematical equivalents.

### 12.2. Should Lp(a) Be Repeated After Acute MI?

As detailed in Section 6, repeat Lp(a) measurement after acute MI should be selective rather than routine and may be considered after clinical stabilization when the initial value is borderline or when precise classification could influence clinical management or trial eligibility [72,73,75,76,77].

### 12.3. What Should Be Done When Lp(a) Is Elevated?

When Lp(a) is elevated, management should focus on guideline-directed secondary prevention and intensive control of modifiable cardiovascular risk factors, as detailed in Section 7. Lp(a)-specific pharmacological therapy should not replace proven interventions in the absence of validated cardiovascular outcome benefit.

### 12.4. Family Implications

Because Lp(a) is strongly heritable, a markedly elevated post-MI result should prompt consideration of cascade screening in first-degree relatives [80], particularly after premature MI or very high Lp(a).

Identifying elevated Lp(a) in asymptomatic relatives may enable earlier modification of conventional cardiovascular risk before clinical ASCVD develops.

### 12.5. When Should Specialist Referral or Trial Participation Be Considered?

Referral to a lipid specialist or preventive cardiology service may be reasonable in patients with markedly elevated Lp(a), premature or recurrent ASCVD despite intensive therapy, familial hypercholesterolemia, complex lipid phenotypes, or uncertainty regarding measurement and interpretation.

Clinical-trial participation is increasingly relevant because ongoing phase III programs evaluating olpasiran, lepodisiran, and muvalaplin use specific Lp(a) eligibility thresholds. Accurate measurement, with confirmation after recovery from acute MI when appropriate, can therefore determine trial eligibility.

### 12.6. What Should Not Currently Be Done?

Current evidence also defines several boundaries for practice. Elevated Lp(a) is not, by itself, an indication for an investigational Lp(a)-specific drug outside an approved indication or clinical trial. A large percentage reduction in Lp(a) should likewise not be treated as a validated surrogate for fewer cardiovascular events; HORIZON illustrates the separation between biomarker and clinical efficacy [16].

### 12.7. A Practical Post-MI Approach

A proposed practical approach to Lp(a) measurement and management after MI is summarized in Figure 3. The framework emphasizes selective repeat measurement after acute MI, intensive secondary prevention, family screening, and consideration of specialist referral or clinical-trial participation in selected patients.

In 2026, elevated Lp(a) after myocardial infarction is best regarded as actionable risk information without an outcome-validated targeted therapy. It can influence the intensity and scope of cardiovascular prevention today, while the role of specific pharmacological lowering remains dependent on future randomized outcome trials.

## 13. Conclusions: From Causal Risk to an Unresolved Therapeutic Question

Lipoprotein(a) [Lp(a)] is firmly established as a genetically determined causal contributor to atherosclerotic cardiovascular disease. This causal role, supported by epidemiological studies, human genetics, Mendelian randomization, mechanistic investigations, and cardiovascular imaging, should be distinguished from its current clinical role after myocardial infarction. In the post-MI setting, Lp(a) presently functions primarily as an inherited marker of residual cardiovascular risk rather than as an outcome-validated therapeutic target. Elevated Lp(a) may identify patients with greater long-term risk of recurrent ischemic events, coronary disease progression, and repeat revascularization, although the magnitude and composition of this risk vary across populations and endpoints.

From a clinical perspective, Lp(a) measurement after MI can refine residual-risk assessment, support cascade screening of first-degree relatives, and reinforce the need for intensive control of modifiable cardiovascular risk factors, particularly LDL-C. When Lp(a) is first measured during the acute infarction and the result is borderline or clinically discordant, selective reassessment after stabilization may improve classification and may also influence eligibility for emerging Lp(a)-lowering trials. However, elevated Lp(a) alone does not currently justify modification of antithrombotic therapy or use of an investigational Lp(a)-specific treatment outside an approved indication or clinical trial.

The sponsor-reported neutral topline result of Lp(a)HORIZON should be interpreted cautiously. It establishes that pelacarsen did not significantly reduce the prespecified primary cardiovascular endpoint in the overall randomized population according to the currently available announcement, but it does not yet explain why the trial was neutral or determine whether effects differed according to baseline Lp(a), absolute reduction, treatment duration, disease stage, individual endpoint components, or prespecified subgroups. These questions should remain open until the complete peer-reviewed results are available.

Several uncertainties therefore remain central to the therapeutic Lp(a) hypothesis. These include the baseline concentration required to identify patients most likely to benefit, the magnitude and duration of absolute Lp(a) reduction needed to influence clinical outcomes, the optimal stage of atherosclerotic disease at which treatment should begin, and whether therapeutic mechanism influences cardiovascular efficacy. Importantly, the profound biomarker reductions achieved with antisense oligonucleotides, small interfering RNAs, and oral inhibitors demonstrate pharmacological target engagement but cannot by themselves be considered evidence of cardiovascular benefit.

Ongoing outcome trials with olpasiran, lepodisiran, and muvalaplin will therefore be critical in determining whether selective pharmacological Lp(a) lowering translates into fewer cardiovascular events and whether clinical benefit depends on patient selection, baseline Lp(a), treatment timing, depth and durability of lowering, or therapeutic mechanism. Until positive randomized outcome evidence becomes available, Lp(a) should be regarded as both a causal cardiovascular risk factor and a clinically useful marker of residual post-MI risk, while its validation as an outcome-improving therapeutic target remains unresolved.

## Figures and Tables

**Figure 1:**
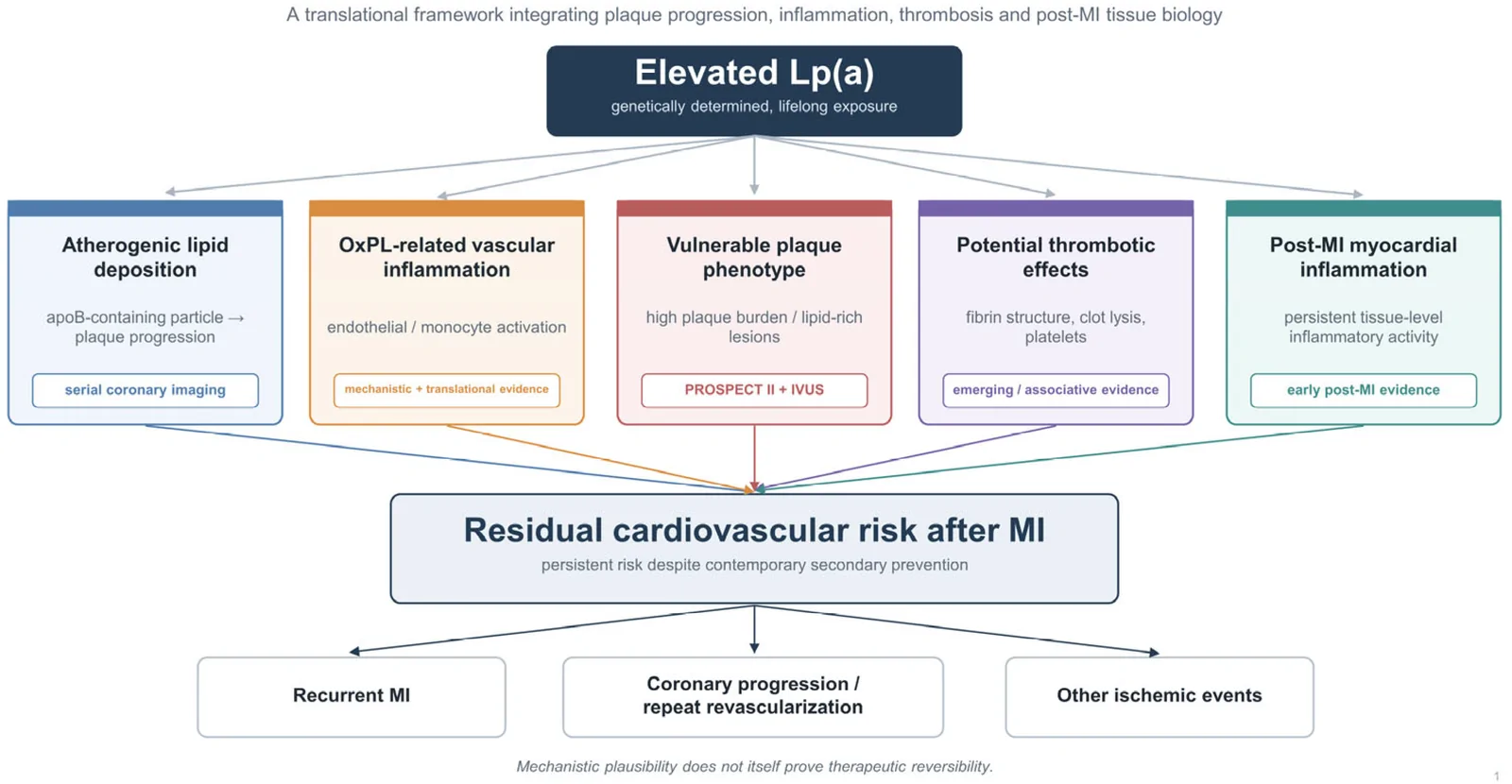
Mechanistic pathways linking elevated lipoprotein(a) [Lp(a)] to residual cardiovascular risk after myocardial infarction. Evidence is most consistent for atherogenic lipid deposition, plaque progression, and adverse plaque characteristics. Oxidized-phospholipid-related vascular inflammation is biologically plausible, whereas thrombotic or antifibrinolytic mechanisms and post-MI myocardial inflammatory activity are supported by less definitive mechanistic, translational, and associative evidence. The figure summarizes biological pathways and does not imply therapeutic reversibility.

**Figure 2:**
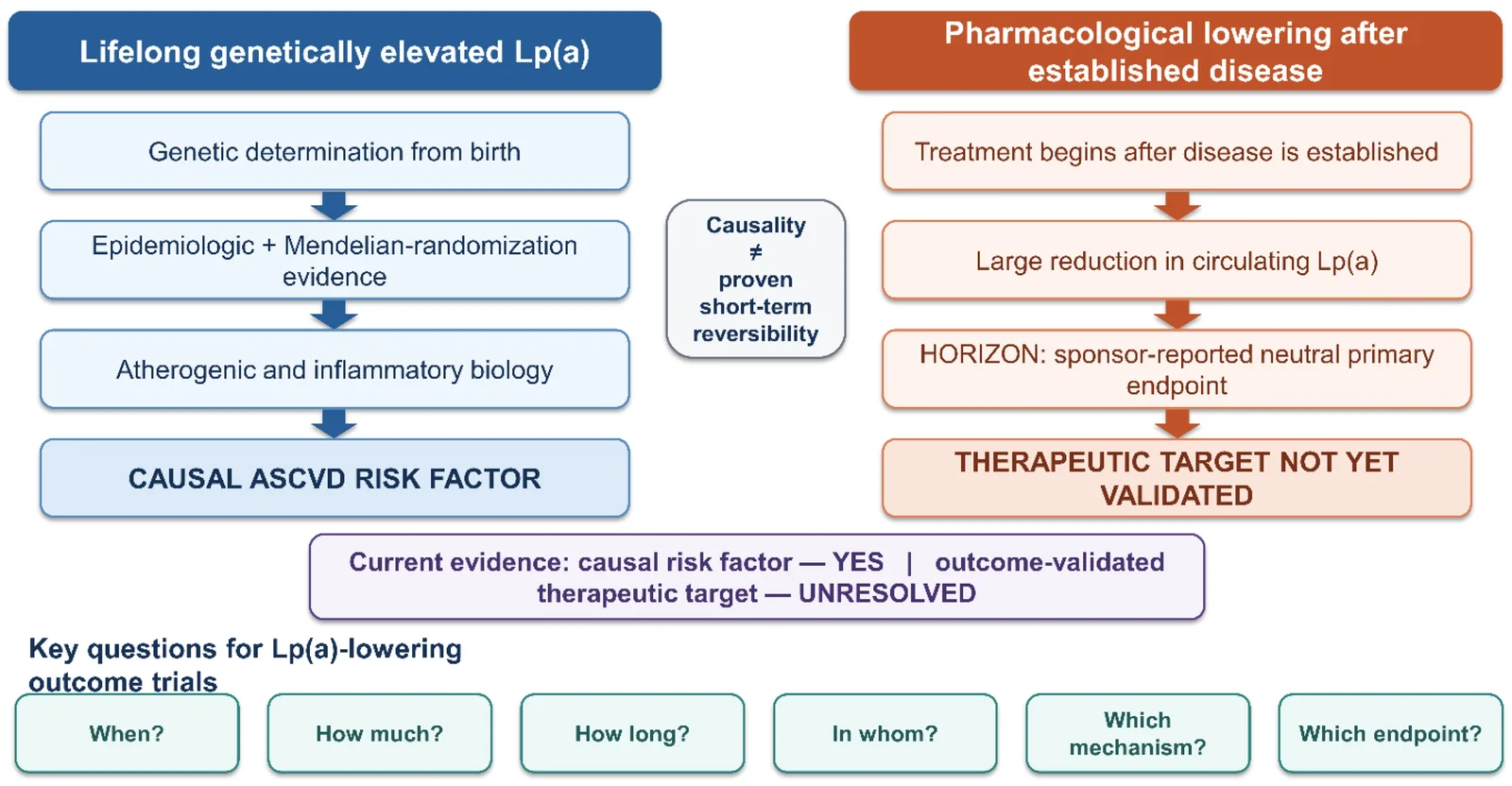
From causal risk to the unresolved therapeutic question: a framework for interpreting lipoprotein(a) after myocardial infarction.

**Figure 3:**
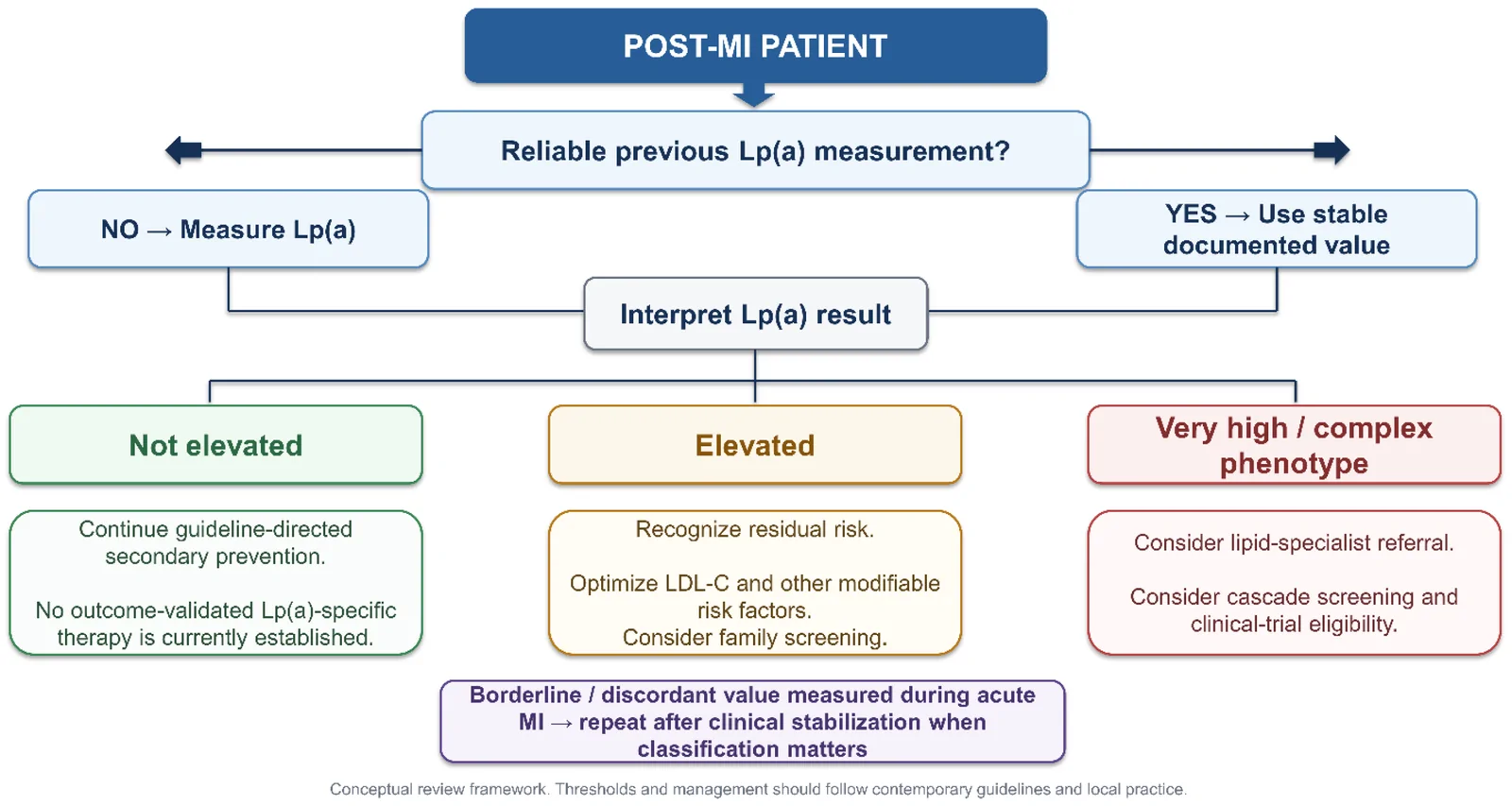
Proposed practical approach to lipoprotein(a) measurement and management after myocardial infarction.

**Table 1:** Representative studies evaluating the association between lipoprotein(a) and recurrent cardiovascular outcomes after myocardial infarction or coronary intervention.

Study	Population	N	Lp(a) Assessment	Follow-Up	Main Endpoint	Main Finding	Key Limitation/Interpretative Point
Cao et al., 2021 [11]	Previous MI	3864	Higher Lp(a); prospective cohort	Median 4.1 years	Recurrent MI, ischemic stroke, cardiac mortality	Higher Lp(a) independently associated with subsequent cardiovascular events.	Observational cohort; residual confounding remains possible.
Liu et al., 2025 [14]	Post-AMI cohorts; meta-analysis	30,027 (23 cohorts)	Categorical and continuous; definitions varied	Variable; stronger association beyond 1 year	MACE	Higher Lp(a) associated with increased post-MI MACE.	Heterogeneity in thresholds, populations, follow-up, and endpoint definitions.
Nishiyama et al., 2026 [56]	STEMI	868	Study-derived threshold: 47.26 mg/dL	Median 736.5 days	CV death, nonfatal MI, nonfatal stroke, any revascularization	Elevated Lp(a) independently predicted the composite endpoint.	Excess risk was driven predominantly by subsequent revascularization.
Miñana et al., 2024 [57]	After acute MI	1223	Continuous/nonlinear relationship	Median ~9.9 years	Recurrent MI; all-cause mortality	Higher Lp(a) associated with greater recurrent MI burden.	No significant association with all-cause mortality.
Šuran et al., 2024 [58]	AMI	2248	>90 mg/dL	Mean 9.5 years	Recurrent MI; CV and all-cause mortality	Lp(a) > 90 mg/dL independently associated with recurrent MI.	No significant CV or all-cause mortality association overall; subgroup signal in women >65 years.
Xu et al., 2022 [59]	Previous CV events undergoing PCI	4469	≥30 mg/dL	~5 years	MACCE; all-cause and cardiac mortality	Elevated Lp(a) independently predicted recurrent cardiovascular events.	PCI population broader than a strictly post-MI cohort.
Yang et al., 2022 [60]	ACS	765	Elevated Lp(a); study-defined	Median 17 months	Recurrent cardiovascular events	Elevated Lp(a) associated with recurrent events.	Clearest component association was with unplanned revascularization.
Cancelli et al., 2025 [62]	Post-PCI; systematic review/meta-analysis	40,241 (14 studies)	Variable across studies	Mean ~4.9 years	MACE, MI, CV death, all-cause death, stroke	Elevated Lp(a) associated with adverse cardiovascular outcomes.	Heterogeneous PCI populations, assays, thresholds, and endpoints.
Wang et al., 2025 [64]	AMI with serial Lp(a) measurements	1131	Admission and 1-year measurement	~50 months	MACCE and individual cardiovascular outcomes	Persistent Lp(a) elevation associated with worse outcomes.	Persistent exposure was defined observationally.
Wang et al., 2025 [65]	AMI	2634	Lp(a) level and cumulative burden	Median 55.2 months	MACCE, CV death, nonfatal MI, nonfatal stroke	Higher Lp(a) and cumulative burden associated with events; strong signal for recurrent nonfatal MI.	Observed cumulative burden is not equivalent to lifelong genetically determined exposure.

Abbreviations: ACS, acute coronary syndrome; AMI, acute myocardial infarction; CV, cardiovascular; Lp(a), lipoprotein(a); MACCE, major adverse cardiac and cerebrovascular events; MACE, major adverse cardiovascular events; MI, myocardial infarction; PCI, percutaneous coronary intervention; STEMI, ST-segment elevation myocardial infarction.

**Table 2:** Targeted lipoprotein(a)-lowering therapies in clinical development.

Agent	Modality/Target	Route	Phase 2 Population	Approximate Lp(a) Reduction	Key Pharmacologic Feature	Outcome Program/ Status
Pelacarsen [102]	Hepatocyte-directed ASO targeting *LPA* mRNA; reduces apo(a) synthesis	SC	*n* = 286; established CVD; Lp(a) ≥ 60 mg/dL	~35–80%, dose-dependent	ASO-mediated apo(a) suppression; q4-week dosing in HORIZON; reduced OxPL-apoB and OxPL-apo(a)	Lp(a)HORIZON [15,16]: phase III; neutral topline primary endpoint
Olpasiran [103]	GalNAc-conjugated siRNA targeting hepatic LPA mRNA	SC	*n* = 281; established ASCVD; Lp(a) > 150 nmol/L	~70.5% to ~97–101% at 36 weeks	Profound, sustained RNA-interference effect; reduced OxPL-apoB [40]	OCEAN(a)-Outcomes [107]: phase III ongoing
Lepodisiran [104]	Long-duration GalNAc-conjugated siRNA targeting LPA mRNA	SC	*n* = 320; elevated Lp(a)	40.8% (16 mg), 75.2% (96 mg), 93.9% (pooled 400 mg); time-averaged days 60–180	Prolonged suppression with potential for infrequent dosing	ACCLAIM-Lp(a) [108]: phase III ongoing
Zerlasiran [105]	Hepatocyte-targeted siRNA suppressing apo(a) synthesis	SC	*n* = 178; stable ASCVD; Lp(a) ≥ 125 nmol/L	~81–86% time-averaged through 36 weeks	Sustained suppression; adverse effects predominantly mild injection-site reactions	No dedicated cardiovascular outcome evidence yet
Muvalaplin [106]	Oral small molecule inhibiting apo(a)-apoB-100 interaction and intact-particle assembly	Oral	*n* = 233; elevated Lp(a)	47.6%, 81.7%, and 85.8% with 10, 60, and 240 mg, respectively	Once-daily oral therapy; assay-dependent interpretation of intact Lp(a)	MOVE-Lp(a) [109]: phase III ongoing

Abbreviations: apo(a), apolipoprotein(a); ASO, antisense oligonucleotide; ASCVD, atherosclerotic cardiovascular disease; CVD, cardiovascular disease; Lp(a), lipoprotein(a); OxPL, oxidized phospholipids; SC, subcutaneous; siRNA, small interfering RNA.

**Table 3:** Major cardiovascular outcome trials evaluating Lp(a)-lowering therapies.

Trial	Agent	Mechanism	Approximate N	Entry Lp(a)	Population	Primary Endpoint
Lp(a)HORIZON	Pelacarsen	ASO	8323	≥70 mg/dL	Established CVD	CV death, MI, stroke, urgent coronary revascularization
OCEAN(a)-Outcomes	Olpasiran	siRNA	7297	≥200 nmol/L	Established coronary ASCVD	CHD death, MI, urgent coronary revascularization
ACCLAIM-Lp(a)	Lepodisiran	Long-duration siRNA	~17,300	≥175 nmol/L	Established ASCVD + high-risk pre-event	MACE-4
MOVE-Lp(a)	Muvalaplin	Oral particle-assembly inhibitor	~10,450	≥175 nmol/L	Previous ASCVD event + high-risk pre-event	MACE-4
OCEAN(a)-PreEvent	Olpasiran	siRNA	~11,000	≥200 nmol/L	No previous major atherothrombotic event	CHD death, MI, urgent coronary revascularization

## Data Availability

No new data were created or analyzed in this study. Data sharing is not applicable to this article.

## Abbreviations

The following abbreviations are used in this manuscript:

ACC/AHA	American College of Cardiology/American Heart Association
ACS	acute coronary syndrome
AMI	acute myocardial infarction
apo(a)	apolipoprotein(a)
apoB	apolipoprotein B
ASCVD	atherosclerotic cardiovascular disease
ASO	antisense oligonucleotide
CHD	coronary heart disease
CVD	cardiovascular disease
ESC/EAS	European Society of Cardiology/European Atherosclerosis Society
LDL-C	low-density lipoprotein cholesterol
Lp(a)	lipoprotein(a)
MACE	major adverse cardiovascular events
MACCE	major adverse cardiac and cerebrovascular events
MI	myocardial infarction
NLA	National Lipid Association
NSTEMI	non-ST-segment elevation myocardial infarction
OxPL	oxidized phospholipids
PCI	percutaneous coronary intervention
siRNA	small interfering RNA
STEMI	ST-segment elevation myocardial infarction
